# Methods of physical treatment for a post-polio adult with scoliosis

**DOI:** 10.1186/1748-7161-7-S1-P25

**Published:** 2012-01-27

**Authors:** B Torres

**Affiliations:** 1 Private Practice, Palo Alto, USA

## Objectives

To describe favorable results in functional postural to enhance and maximize lumbar lordosis at L1/L2 level as well as thoracic kyphosis, increasing the possibility of correcting the postural part of scoliosis in 3D and adding ergonomics.

## Case report

A 69 years old female with 85° left thoracic scoliosis and bone fusion surgery at 19 years old at T10/T12 level with onset of pain and disability following menopause. The patient had chronic pains as measured on the Roland and Morris VRS as 4 (very strong).

## Methods

The ScoliologiC “Best Practice” program with physi-logic exercises, correction of ADL (activities of daily living), 3D- made easy with “ maximum “ possible corrections for the past two years and Lehnert-Schroth exercises, starting seven years ago.

## Results

The patient is able to maximize corrections, free of pain during standing, walking, exercising for 1/2 hour, sitting and resting on bed using proper ergonomics. Roland and Morris VRS value actually in 0-1 (no pain/ little pain).

## Conclusions

With thoughtful and reasonable organized physical activities, the patient is able to control her physiological and scoliotic curves, preventing pain, improving the cosmetics, vitality, endurance, functional activities and well being.
